# Patient adherence to prescribed artemisinin-based combination therapy in Garissa County, Kenya, after three years of health care in a conflict setting

**DOI:** 10.1186/s12936-015-0645-z

**Published:** 2015-03-24

**Authors:** Georgia R Gore-Langton, Nfornuh Alenwi, James Mungai, Nahashon I Erupe, Katie Eves, Francis Njoroge Kimwana, David Soti, Willis Akhwale, Farah A Hassan, Elizabeth Juma, Richard Allan

**Affiliations:** The MENTOR Initiative, Crawley, UK; The MENTOR Initiative, Garissa, Kenya; Ministry of Health, Garissa, Kenya; Ministry of Health, Nairobi, Kenya

**Keywords:** Malaria, ACT, Adherence, Conflict setting, Kenya

## Abstract

**Background:**

Current day malaria cases and deaths are indicative of a lack of access to both methods of prevention, diagnosis, and treatment; an important determinant of treatment efficacy is adherence. This study is a follow up to the baseline study of adherence to artemether-lumefantrine (AL) carried out in Garissa District in 2010. The study presented evaluates any changes in adherence levels which may have occurred in the area during this period and after nearly three years of sustained use of ACT across the public health sector.

**Methods:**

The study was carried out in Garissa County in the North Eastern Province of Kenya and included patients fitting the suspected malaria case definition and having been prescribed AL, regardless of confirmatory diagnosis. A questionnaire assessed the intake of AL via both self-reporting by the participant and observation of blister packs by the interviewer. On separate occasions exit interviews with patients and observations of prescribers were also carried out.

**Results:**

Of the 218 participants enrolled, 195 were successfully followed up. 60% of participants were found to be adherent to the three-day AL regimen, this is 4.7% lower than the proportion of participants adherent in 2010; the result of a two-sided z-test was not significant (p = 0.23). The odds of the patient being adherent to AL increased by 65% with each additional correct statement regarding how to take AL that a patient could recall (between zero and four statements), this was the only variable significantly associated with patient adherence (p = 0.01).

**Conclusion:**

Sustaining the ACT adherence rates at the 2010 levels, through 2.5 years of insecurity in the study area is an achievement and suggests that if security can be improved barriers to improving health service quality and patient adherence to AL would be removed. This study, by looking specifically at anti-malarial adherence over a prolonged period and in a setting of severe conflict, provides a valuable and rare insight in to the challenges and barriers to ACT adherence in such settings.

## Background

There were 198 million (range 124–283) cases of malaria and 584,000 (range 367,000- 755,000) deaths from malaria in 2013. These large figures are a considerable decrease on the 2000 levels since when malaria cases have dropped by 26% worldwide, and the number of deaths due to malaria has fallen by 47% worldwide [1]. These reductions in morbidity and mortality are testament to continuing efforts to improve access to key malaria control interventions - LLINs, malaria diagnostics and effective anti-malarials. The use of anti-malarials has a long history, from the bark of Chinchona trees containing quinine isolated in 1820, to the development of chloroquine and then chloroquine-resistant *Plasmodium falciparum* [2]*.* It was as part of the search for new and safer anti-malarials that artemisinin-based drugs emerged from China, becoming widely available outside of China for the first time in the 1990s. With the hope of preserving the efficacy of this anti-malarial it is recommended only in combined use with other anti-malarial, as artemisinin combination therapy (ACT) [3]. Between 2003 and 2010 all malaria endemic countries in Africa switched to using ACT as a first-line treatment for uncomplicated malaria.

ACT has a more complicated dosing regimen than monotherapies, in the case of artemether-lumefantrine (AL) this ranges from one to four tablets (determined by a patient’s weight) taken twice a day for three day. The efficacy of ACT is very high, however, this is assuming a patient is adherent to the complete course of treatment [4-7]. The definition of full adherence differs between studies, in this study self-reporting to have correctly taken the entire course of treatment was the definition used. Regardless of how defined, adherence is determined and affected by numerous and intertwining factors including age of the caretaker and patient, education level of the caretaker and patient, perceptions of the disease, acceptance and knowledge of the treatment, cost of treatment, complexity of the treatment schedule, quality of prescription, and the patient’s clinical improvement, with these factors being associated with either an increased or decreased likelihood of being adherent [8-10]. The consequences of poor patient adherence are serious; cure rates for patients are likely to be lower, and repeat exposure to sub-lethal drug dosages risks the development of parasite resistance to the active agents in the drug formulations [11,12].

In Kenya, *P. falciparum* accounts for almost 100% of malaria infections [1]; the main vectors are *Anopheles gambiae, Anopheles arabiensis,* and *Anopheles funestus* [13]*.* Extrapolating on the 2012 Kenyan population estimate of 43.18 million with the yearly growth rate of 2.7% [14], the estimated population in 2014 is 45.54 million. 76% of the population live in areas of malaria transmission (36% in areas of high transmission, 40% in areas of low transmission) [15]. In 2013 there were 2, 335, 286 confirmed cases of malaria in Kenya [1]. ACT in the form of AL is the first-line treatment for uncomplicated malaria in Kenya. AL was rolled out in 2006 after the very sudden decline in efficacy of sulphadoxine-pyrimethamine (SP) [16].

The baseline study carried out by The MENTOR Initiative in 2010 in Garissa County, North Eastern Province, Kenya, found that of a sample of 272 patients, 176 (64.7%) were “probably adherent”, defined as patients claiming to have completed all doses whether or not they were able to show the empty blister packet. The strongest predictor of adherence was found to be patient knowledge of the ACT regimen, if a patient was able to cite at least one correct instruction as to how to take AL they had 1.76 (95% CI: 1.32, 2.35, p < 0.0001), the odds of being fully adherent compared to a respondent that could not cite any instructions [17]. This study noted the importance of patient awareness of the AL dosing regimen and focusing on health worker training and prescribing practises.

Since the baseline study in 2010 The MENTOR Initiative has supported the Ministry of Health (MoH) to reinforce malaria case management in Garissa, through the introduction of rapid diagnostic testing (RDT) for patients with suspected malaria, prior to treatment. Health workers received regular technical coaching to encourage them to explain the AL treatment regime to patients and there was a comprehensive package of malaria prevention activities. In addition, and in line with the 2011 study recommendations [17], Information, Education, and Communication (IEC) and Behaviour Change Communication (BCC) campaigns have been run in the study area. Materials such as radio spots, posters, drama campaigns, and billboards and mural paintings have been used to strengthen community awareness on malaria prevention and treatment, including the importance of adherence to the full course of ACT.

The purpose of this study is to measure current day patient adherence to the three day AL treatment regimen, and determine any changes in adherence since the 2010 study. Whilst the 2010 study was conducted in the areas of Bunyala, in Western Kenya, and Garissa, this 2013 study included only the area of Garissa; for this reason all comparative analysis between the two studies shall only take in to account the 2010 data collected in Garissa.

## Methods

### Study area

**Figure 1:**
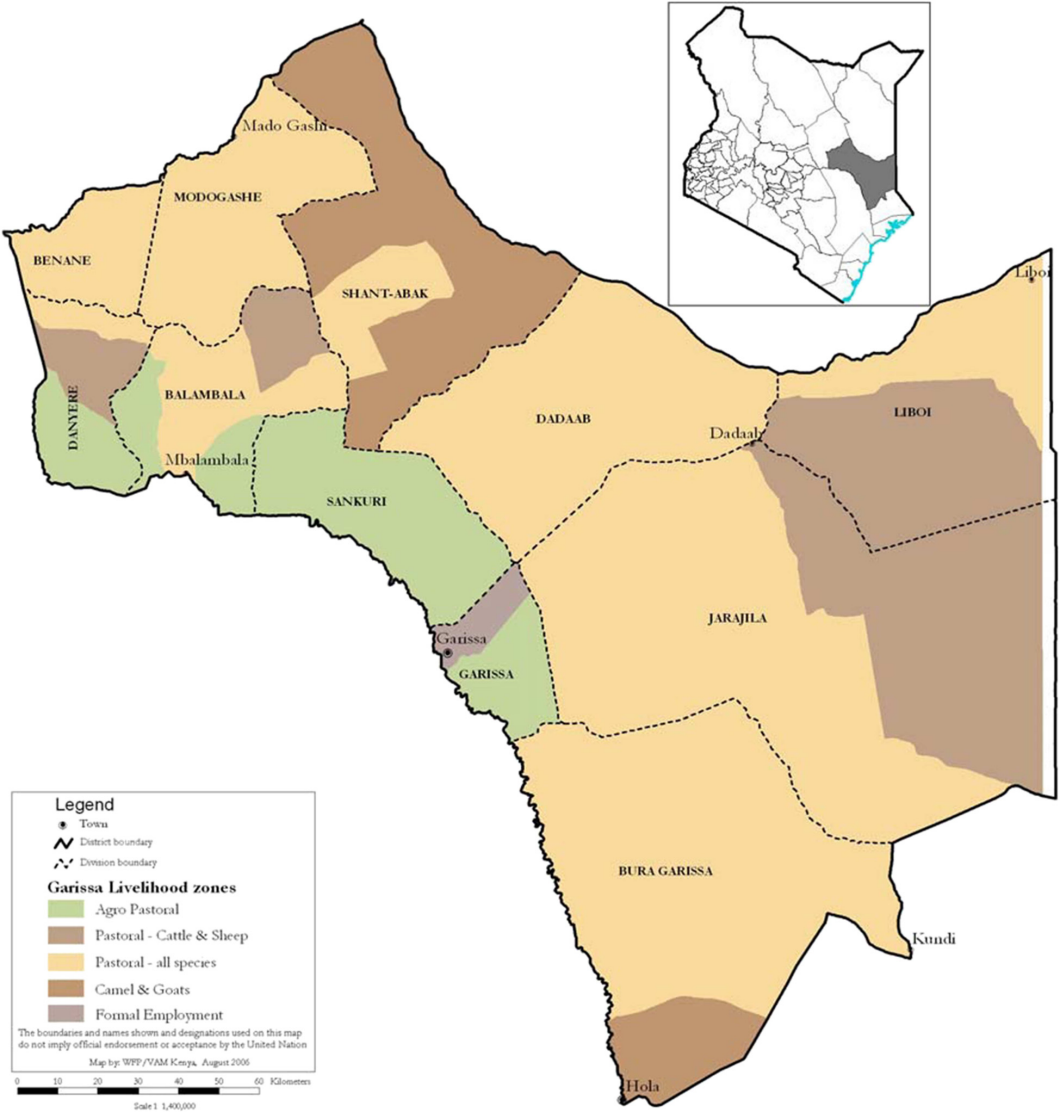
**Map of Garissa County, Kenya.**

Garissa County is located in what was formerly the North Eastern Province of Kenya and covers an area of 44,175 square kilometres (km^2^). It borders Wajir County to the North, Tana River County to the West, Lamu County to the South, and Isiolo to the North West and shares an international boundary with Somalia to the East (Figure 1). The population is 95% ethnic Somali and according to the last national census in 2009 was 623,060 [18]; expected to have grown to 715, 312 in 2014 based on the UNICEF prediction of a 2.8% annual growth rate [19].

Garissa County comprises six districts namely Garissa, Balambala, Lagdera, Ijara, Dadaab, and Fafi. There are 62 public and private health facilities in Garissa County, including those serving Dadaab Refugee Camp. The climate is semi-arid with a range in temperature from 21°C to 39°C in 2012 [20] and an annual average bimodal rainfall (rainy seasons from March – May and September – October) of 250 – 300 mm. Data collection started in June 2013 and continued through to the end of October 2013, overlapping with the long and often heavy rainfall from September onwards.

Malaria transmission in Garissa is strongly seasonal; low during most of the year but epidemic prone during the rainy season [21]. In 2013, there were 4,519 clinical cases of malaria and 1,321 confirmed cases of malaria in Garissa, this is a 92% reduction in both clinical and diagnosed malaria cases in the area since 2010 (unpublished Kenyan Ministry of Health data). The primary vectors in the region are mainly exophagic and exophilic, such as *An. coustani* and *An. arabiensis* [22-24]*.* Both *P. falciparum* and *P. vivax* are present with *P. falciparum* accounting for the vast majority of cases [1,25]. Historically, due to poor understanding that not all fevers are malaria and that malaria is seasonal, and a general lack of confidence in health systems (especially among the ethnic Somali communities), malaria has been greatly over-diagnosed in this area. Diagnostic tools have either been unavailable, misused, negative results not believed, or diagnosis has been based on clinical symptoms alone [26].

### Study population

All study participants were patients whose symptoms fitted the definition of a suspected malaria case: fever (axillary temperature ≥37.5°C) or history of fever in the last 48 hours with other obvious causes of fever excluded (typhoid, yellow fever, dengue fever, diphtheria, Rift Valley fever, among others [27,28]), patients considered for a malaria diagnostic test, weight ≥5 kg, age >5 months, and with no signs of complicated malaria or other severe disease. Those who fitted this definition and were treated with AL were included as a study subject regardless of whether or not the diagnosis was confirmed. Patients were excluded from the study if they had suffered from a separate episode of malaria in the previous two weeks, or if they or a household member had previously participated in the study, this ensured that no family was interviewed twice.

### Study procedure

Six health facilities in Garissa were involved in the study, three of which had participated in 2010. Health workers at the health facilities informed their patients to keep the empty AL blister packs after treatment had been completed. Patients were not told why they were being asked to keep the blister packs in order to maintain the validity of the results. At the end of each day, the registers of the health facilities were consulted to identify patients who met inclusion criteria, these patients or their caretakers were visited at home by the study team and community health workers on the fourth day after receiving treatment.

At the beginning of the household visit, written consent was requested from each adult patient, and the parent or guardian of every child patient included in the study. The patient’s axillary temperature was taken. A structured questionnaire – available in English and translated into Somali and field-tested – was given to the participants. The questionnaire assessed the intake of AL via both self-reporting by the participant and observation of blister packs by the interviewer. Socio-demographic information on the patient was collected and the patient or caretaker’s language, age and education level, the number of people who spent the previous night at the house, and the number of children cared for were all recorded.

The questionnaire was concluded by counting any remaining tablets. In cases where tablets remained, patients/caretakers were asked to specify why not all tablets were taken by the patient/ given to the child. Questions were asked in such a way that honest answers were encouraged, without placing blame on interviewees. The team of interviewers were supervised by MENTOR Initiative clinical staff.

Patients were categorized as definitely non-adherent; probably non-adherent; and probably adherent based on the following classifications. Those who presented blister packs with AL doses still remaining were definitely non-adherent; patients or caregivers who claimed doses not completed but who did not show a blister pack were classified as probably non-adherent; those who claimed to have completed all doses and could show an empty blister pack were classified as probably adherent as were patients who could not show an empty blister pack but claimed to have taken all of the tablets. Non-adherent patients were advised to return to the health clinic for a new assessment to avoid negative consequences from an incomplete course of treatment.

In order to identify possible factors influencing adherence, two additional investigations were carried out. The week after the completion of collection of adherence data, patients or their caretakers were systematically interviewed after leaving the pharmacy (out of sight of the pharmacy in order to reduce bias) to assess how well they had understood the AL prescription. A short questionnaire, asking for a spontaneous account of how the treatment was to be taken/ given to the child, was carried out followed by systematic questioning for each treatment dose.

On separate occasions the pharmacists or other persons dispensing medication in each health facility were systematically observed while explaining the AL prescription to patients or caretakers. A check-list was used to verify what information was given: *e.g.* timing of drug intake, importance of completing the treatment. The observers stood next to the patient during the explanation so the pharmacist/person dispensing the medication knew a study was being conducted, but he/she was blinded as to the purpose of the observation.

### Analysis

Open-ended questions were reviewed and grouped into thematic categories that were coded for data entry. Results were double entered into Epi Info 3.5.1 (CDC, WHO) and all data were analysed in STATA 13.0 (Stata Corporation, College Station, Texas). Data were summarized using proportions and means and medians, where appropriate. Comparisons of proportions between categorical variables were made using two-tailed Z-tests. Univariate and multivariate logistic regression was used to test for significance between potential explanatory variables and the dichotomous outcome variable of the patient being adherent or not. Significance tests were determined at the 10% level.

### Ethical consideration

The study was not an intervention study and was not designed to influence the diagnosis and treatment of the participants. Permission to carry out the study was gained from the University of Nairobi, The Ministry of Health, Kenya, and Kenya National Hospital ethics review committee.

At the beginning of the visit written consent was requested from every adult and parent or guardian of every child considered for inclusion in the study.

## Results

### Description statistics

A total of 218 respondents were enrolled in the study. They came from six health facilities from within Garissa district (Garissa town, capital of Garissa County). Of these enrolled participants 23 (10.5%) were either lost to follow-up (n = 19) or didn’t respond to later questioning (n = 4), bringing the total number of participants successfully followed-up down to 195. The majority of participants were male (81.0%) and over 15 years of age (99.0%), half of participants owned their own businesses (53.8%), when asked about education 32.3% reported never having attended a school or college, just over half of all households contained 0–5 people (55.4%) with the remaining containing six or more people. Characteristics of the study population are shown in Table 1.

**Table 1:** **Descriptive characteristics of the study population**

**Variable**	**N**	**% (95% CI)**
**Patient sex**		
Male	158	81.0 (74.8, 86.0)
Female	37	19.0 (14.0, 25.2)
**Patient age**		
≤15 years	2	1.0 (0.25, 4.1)
>15 years	193	99.0 (95.9, 99.7)
**Patient education**		
Never attended	63	32.3 (26.1, 39.2)
Primary	55	28.2 (22.2, 35.0)
Secondary	48	24.6 (19.0, 31.2)
Post-secondary	25	12.8 (8.8, 18.3)
Not applicable	4	2.1 (0.8, 5.4)
**Head of household’s job**		
Farming	1	0.5 (−0.4, 1.4)
Own Business	105	53.8 (47.2, 60.4)
Retired	1	0.5 (−0.4, 1.4)
In school	2	1.0 (−0.3, 2.3)
Other	50	25.6 (19.8, 31.4)
Unemployed	36	18.5 (13.4, 23.7)
**Household size**		
0-5 people	108	55.4 (48.3, 62.3)
≥6 people	87	44.6 (37.7, 51.7)

### Patient adherence

Of the 195 participants successfully followed up, 60.0% were found to be probably adherent to the three day ACT regimen. Of the 78 participants classified as non-adherent, 31 (39.7%) were definitely non-adherent and 47 (60.3%) were probably non-adherent. This proportion of patients found to be probably adherent in 2013 is 4.7% lower than the proportion of participants probably adherent in 2010. The result of a two-sided z-test between the proportion adherent in 2010 and 2013 was not significant at the p ≤ 0.05 level (Table 2).

**Table 2:** **Comparative analysis of adherence in Garissa in 2010 and 2013**

**Year**	**Total participants**	**Probably adherent**	**% probably adherent (95% CI)**	
2010	272	176	64.7 (59.0 70.4)	p = 0.23
2013	195	117	60.0 (52.9, 66.7)	

Other notable differences between the study populations include differences in the sex and age of participants. In the 2010 study population of Garissa the proportion of female to male participants was greater than it was in 2013; in 2010 there were 160 females (55.8%) and 112 males (44.2%), in 2013 there were only 37 females (19.0%) and 158 males (81.0%). In 2013 the proportion adherent differed slightly between male and female, 59.5% probably adherent and 62.2% probably adherent, respectively. The 2010 study population also contained many more participants under the age of 15 years. In 2013 only two study participants (1%) were children under 15 years of age compared to the 136 participants (50%) in the 2010 study population in Garissa. Of the 2013 study population the majority tested RDT positive for malaria (80.5%) with 37 participants testing negative (18.9%), one participant did not know their RDT result (0.5%).

Univariate logistic regression compared 17 variables thought to be potential predictors of patient adherence. Of these 17 variables, 11 were not fitted in to a multivariate logistic regression model as their crude (bivariate) odds ratios were not significant (p ≥ 0.10); one variable- whether or not the patient had difficulty in understanding the prescription instructions- was excluded as collinearity with adherence was shown. The remaining five variables with p-values ≤0.10 were fitted in to the multivariate logistic regression model (Table 3). The only variable significantly associated with patient adherent to a course of AL treatment in the multivariate model was the number of correct statements regarding how to take AL that the patient could recall (p = 0.01). The odds of the patient being adherent to AL increased by 65% with each additional statement they could recall (between zero and four statements).

**Table 3:** **Bivariate (crude) and multivariate (adjusted) model of association between adherence and exposure variables**

**Variable**	**Adherent (%)**	**Non adherent (%)**	**Crude odds ratio (95% CI)**	**P-value**	**Adjusted odds ratio (95% CI)**	**P-value**
**Patient education**				0.06		0.18
Never attended	29 (46.0)	34 (54.0)	1		1	
Primary	40 (72.7)	15 (27.3)	3.13 (1.44, 6.77)		2.44 (1.05, 5.67)	
Secondary	30 (62.5)	18 (37.5)	1.95 (0.91, 4.20)		2.06 (0.90, 4.72)	
Post-secondary	16 (64.0)	9 (36.0)	2.08 (0.80, 5.42)		2.84 (0.96, 8.46)	
Not applicable	2 (50.0)	2 (50.0)	1.17 (0.16, 8.85)		1.55 (0.10, 24.60)	
**Price paid for treatment**				0.05		0.07
Nothing	98 (63.6)	56 (36.4)	1		1	
10 Kenyan Shillings	1 (100)	0	-		-	
Other Amount	17 (46.0)	20 (54.1)	0.49 (0.24, 1.00)		0.46 (0.21, 1.02)	
Don’t know	1 (33.3)	2 (66.7)	-		-	
**Who provided treatment**				0.04		0.79
Clinic/HF pharmacist	104 (63.8)	59 (36.2)	1		1	
Doctor/ clinical officer/ nurse	9 (36.0)	16 (64.0)	0.32 (0.13, 0.77)		0.65 (0.05, 8.47)	
Private pharmacy	0	1 (100)	-		-	
Community Health Worker	3 (60.0)	2 (40.0)	0.85 (0.14, 5.24)		0.44 (0.03, 6.15)	
Don’t know	1 (100)	0	-		-	
**Who explained how to take**				0.01		0.62
Clinic/HF pharmacist	105 (63.3)	61 (36.8)	1		1	
Doctor/ clinical officer/ nurse	7 (33.3)	14 (66.7)	0.29 (0.11, 0.76)		0.50 (0.03, 7.77)	
Private pharmacy	0	1 (100)	-		-	
Family member/friend	1 (100)	0	-		-	
Community Health Worker	1 (33.3)	2 (66.7)	0.29 (0.3, 3.27)		-	
Other	2 (100)	0	-		-	
**Number of statements recalled by patient**				0.002		0.01
Each additional correct statement recalled by patient (0–4)	117 (60.0)	78 (40.0)	1.78 (1.25, 2.54)		1.65 (1.10, 2.46)	

### Patient’s knowledge and perceptions

The follow up questionnaire/survey of patients four days after their visit to the health facility and their being prescribed ACT collected information on their knowledge and perceptions regarding malaria and its treatment. Table 4 shows that the majority (77.4%) of patients had waited less than a day from when first experiencing symptoms before visiting a health facility. Nearly all patients (93.3%) reported having seen AL before and more than half had taken AL before (64.3%). Only a small proportion of patients reported disliking AL (3.1%) while a fifth (21.5%) reported side-effects to the treatment. Of side effects reported, the most common was vomiting (82.6%) followed by fever (10.9%). When asked about prescription practices, 95.4% of patients could report at least one correct statement while 53.9% could report at least two or more statements; correct statements included the timing of taking doses, taking doses with food or milk, and returning to a health facility if condition deteriorated. Just over half of patients (56.7%) reported not to have had malaria in the last month. Almost all patients (98.5%) reported having had an RDT test, of these 80.5% reported testing positive for malaria.

**Table 4:** **Patients malaria knowledge and perceptions**

**Variables**	**N**	**% (95% CI)**
**Days waited before visiting health facility**		
≤1 day	151	77.4 (71.0, 82.8)
>1 day	44	22.6 (17.2, 29.0)
**Patient Seen AL before**		
Yes	182	93.3 (88.8, 96.1)
No	12	6.2 (3.5, 10.6)
Don’t Know	1	0.5 (0.1, 3.6)
**Patient Taken AL before**		
Yes	63	64.3 (54.2, 73.3)
No	35	35.7 (26.7, 45.8)
**Dislike AL**		
Yes	6	3.1 (1.4, 6.7)
No	189	96.9 (93.3, 98.6)
**Side Effects**		
Yes	42	21.5 (16.3, 27.9)
No	153	78.5 (72.1, 83.7)
Fever	5	10.9 (1.9, 19.9)
Vomiting	38	82.6 (71.6, 93.6)
Headache	2	4.3 (−1.6, 10.6)
Weak & dizzy	1	2.1 (−2.0, 6.2)
**Number of correct statements recalled**		
None	9	4.6 (2.4, 8.7)
At least one	186	95.4 (91.3, 97.6)
**Last time patient had malaria**		
≤1 month ago	88	43.4 (36.6, 50.3)
>1 month ago	115	56.7 (49.7, 63.4)
**Did patient receive RDT**		
Yes	192	98.5 (95.3, 99.5)
No	0	0
Don’t know	3	1.5 (0.5, 4.7)
**RDT Result**		
Negative	37	18.9 (14.0, 25.2)
Positive	157	80.5 (74.3, 85.5)
Don’t know	1	0.5 (0.07, 3.6)

### Observations and exit interview questionnaire

Data collected via both observations of prescriptions and exit interviews (Table 5) showed taking doses in the morning and evening for the two days after the first dose is the message most frequently given to patients. Repeating doses in the case of the patient vomiting within 30 minutes of taking the original dose was the least frequently stated message in both contexts.

**Table 5:** **Data collected via observation and exit interviews about the messages given to patients by prescribers**

	**Data collected via observations of prescription n = 90**		**Data collected via exit interviews n = 89**	
**Message given to patient**	**N**	**% (95% CI)**	**N**	**% (95% CI)**
Take first dose straight away	68	75.6 (65.4, 83.5)	68	76.4 (66.3, 84.0)
Take second dose 8 hours after first	56	62.2 (51.6, 71.8)	31	34.8 (25.5, 45.5)
Take remaining doses in morning and evening for 2 days	70	77.8 (67.8, 85.3)	83	93.3 (85.6, 97.0)
All three above messages on timings	44	48.9 (38.6, 59.3)	31	34.8 (25.5, 45.5)
Take tablets for 3 days	63	70 (59.6, 78.7)	-	-
Take all the tablets	62	68.9 (58.4, 77.7)	79	88.8 (80.2, 93.9)
Take with food/milk	19	21.1 (13.8, 31.0)	17	19.1 (12.1, 28.8)
Repeat dose if vomit within 30 minutes	12	13.3 (7.6, 22.2)	9	10.1 (5.3, 18.5)
Return to health facility if deteriorate	17	18.9 (4.1, 12.0)	19	21.4 (13.9, 31.3)

## Discussion

The ACT adherence study carried out in 2010 in Garissa and Bunyala was the first of its kind in Kenya and was important in informing the Ministry of Health nationally, and regionally, on the success of the introduction of ACT since 2006, and the factors associated with either high or low levels of adherence [17]. The follow-up study carried out in Garissa in 2013 and presented here allows for comparisons of ACT adherence levels three years on and after a period of consistent activity by the MoH aimed at consolidating and improving the quality of malaria case management services across Garissa.

Similar studies in to the adherence of patients to different treatment regimens for uncomplicated malaria show varying results. Using self-reporting, pill counts, and blood drug levels to investigate the adherence of patients to a six dose regimen of AL in Uganda found 90% of patients to be probably adherent [29]. In the Maheba refugee settlement of Zambia, 39.4% of patients were classified as probably adherent to a three-day course of the combination of artesunate and SP, 21.2% were certainly non-adherent, and 39.4% were probably non-adherent [30].

The 4.7% decrease in proportion adherent in Garissa from the 2010 level of 64.7% is not statistically significant and so should be interpreted as no significant change in adherence levels between the years, rather than a true decrease. This result is disappointing, however, the lack of apparent improvement does not mean the data is of no interest, nor does it reflect the improvements seen in the uptake and usage of RDTs and ACT by health workers across the region [26]. Possible explanations for an unchanged level of adherence include limitations to the study design and differences between the two study populations in 2010 and 2013, contextual reasons outside the control of the programme and/or flaws and/or oversights in the efforts to achieve behaviour change during the intervention period.

Differences in the study populations between 2010 and 2013 in Garissa may have impacted the adherence levels seen. The sample size of patients in 2013 (n = 195) was much smaller than the sample size in the 2010 study (n = 918) [17]. This drop in patient numbers is due in part to the reduced sampling area in 2013, the exclusion of Bunyala which was sampled in 2010, and also to the great reduction in the number of clinical and confirmed malaria cases over the three year period (both reduced by 92%). Whilst this reduced sample size may have limited the power of the study it is great testament to malaria control and prevention work carried out in the area. There were also changes in sex and age of study participants between 2010 and 2013. The proportion of male participants was far greater in 2013 than in 2010 (81.0% compared to 41.2%, respectively) and the proportion of study participants under the age of 15 years was much smaller in 2013 compared to 2010 (1% compared to 50%, respectively). Although neither sex nor patient age were shown to be statistically associated with adherence, differences such as these in the study populations are potential limitations to comparative analysis between adherence levels in 2010 and 2013.

The methods used to measure adherence and the categorisation of patients as adherent or not differ between adherence studies. The majority of methods can be summarised with five overarching approaches; “Completed treatment” identifying individuals who say they completed treatment, “verified completed treatment” corroboration of reported completed treatment with a pill count, “timely completion” refers to patients reporting timely completion, “verified timely completion” refers to those reporting timely completion and with a pill count to confirm no tablet left. The last method is “biological assay” which uses the detection of sufficient levels of the drug in biological samples to confirm adherence [10]. The method used in this study- verified completed treatment- does not attempt to determine whether or not the patient took the AL doses at the correct timings, simply whether or not the complete course of treatment was taken. Whilst this method is vulnerable to misreporting by patients and skewing of results in ways that biological assays are not, it has been used in similar adherence studies to obtain realistic and valuable results [17,31,32].

While policy is to give ACT for free at health facilities, the study has shown that this is often not the case. 37 patients (19%) reported paying for their treatment; the largest proportion of these paid 100 Kenyan Shillings (KSH) (n = 22, 59.5%), equivalent to one United States Dollar (USD), five people paid less, and nine paid more. The highest price reported was 800 KSH. Of those reportedly paying for treatment, 83.8% (n = 31) received treatment from a clinical pharmacist, 13.5% (n = 5) received treatment from a doctor, or nurse, and 2.7% (n = 1) received treatment from a private pharmacy. The proportion of Kenyans living below the international poverty line of 1.25 USD per day (roughly 110 KSH) is 43.4% [33]. Having to pay even 30 KSH (the lowest price reported), let alone 100 KSH or above, is a significant proportion of this total daily allowance. While this evidently hasn’t been a deterrent to seeking health care among this study population - participants are patients who have been prescribed AL and paid the costs asked for – it is possible that care takers and patients that have to pay for treatment may sub-divide treatments between multiple family members, or save part of the treatment for future malaria cases in the family [34,35]. It is feasible that either patients do not trust the dosing advice given by prescribers once they learn they have been misinformed about the price of ACT; or that they in fact pay more attention to the advice of the prescriber purely because they are paying for the treatment. Whilst it was not shown in this study that the price of treatment was associated with patient adherence, the issue of pricing is one that should be addressed in order to ensure free malaria diagnosis and treatment is available to all, from prescribers trusted by the patient.

Interventions aiming to improve patient adherence to a treatment regime have been numerous and varied. From improving adherence to short-term treatments, such as anti-malarials, to treatments for chronic conditions, such as hypertension, various methods have been investigated including changing dosing schedules (for example, from two tablets a day to one), reminder pill packaging, individual counselling, home visits by research assistants, and reward schemes for high adherence [36,37]. A study in Ghana found that increased provision of information to clients by providers in primary health care facilities resulted in increased levels of adherence; improvements were most marked in the clinic that was found to be worst performing at the beginning of the study [38]. A BCC intervention in Kenya, which involved the training of drug vendors to treat childhood fevers with chloroquine and provided age-specific dosing charts saw adherence to the correct age-specific dose increase from 4% to 75% and correct duration from 23% to 47% [39]. Studies and results of this nature provide information on how to tailor IEC/BCC campaigns towards specific communities and contexts.

In the study area of Garissa the ethnic Somali population is a marginalized one anecdotally sceptical of outside interventions and IEC campaigns, making the challenge of real behaviour change even greater. The number of correct statements reported by the patient was the most significant variable in predicting adherence in this survey and the 2010 survey [17], this result emphasises the important of patient IEC campaigns and the clear and correct relay of information from the prescriber to the patient. Results from exit interviews and prescription observations showing that the majority of treatment regime messages are being given to more than half of patients suggest that many patients have at least some knowledge of how to correctly take AL treatment; considered alongside the relatively low adherence rate of 60% this suggests that IEC campaigns must go along side campaigns informing patients of the important and benefits of being adherent and encouraging them to act on the knowledge they have.

Local contextual factors are also likely to have had an effect on adherence levels. Security in Garissa has historically been fragile. From 2010 onwards conflict inside Somalia intensified resulting in significant influxes of new refugees into the camps in Garissa Town. In October 2011, Kenya sent 10,000 troops into southern Somalia, largely through Garissa, to attack and clear Al Shabaab terrorist groups that controlled areas neighbouring Garissa [40,41]. The Kenyan military presence in South Central Somalia has been maintained since that time as part of an international effort to bring peace to the country. One side effect of the ongoing Kenyan military operation into southern Somalia has been a dramatic escalation of Al Shabaab incursions into Garissa County and attacks on the Kenyan public [42]. Whilst Garissa Town has suffered only sporadic attacks and has been the least affected area in the county, any increases in instability are likely to have had indirect effects on health services. The Somali population in the area, likely to have made up the largest proportion of the study participants, are anecdotally untrusting of outside intervention, including the health care system. As results have shown education levels in the area are very low and are likely to greatly impact understanding and knowledge of malaria which is shown to be a significant predictor of adherence. The insecurity has also meant that staff turnover in health facilities has been very high and the facilities typically do not attract the highest quality of staff, again likely to comprise the clarity and quality of health care given to patients, including AL treatment regime information. In this particular setting, conflict is suspected to have had an impact both on the ability of the health care system and health care workers to sustain the quality of services provided in times of peace. Achieving patient behaviour change in settings such as these when the priorities of communities are focused largely on survival and coping mechanisms becomes an even greater challenge.

## Conclusion

This paper has laid out the findings of an ACT adherence study in Garissa County, Kenya, nearly three years after a base-line study and efforts to improve ACT prescription practices and adherence. Results showed no significant change in adherence levels over the period. The ability of patients to recall prescription practise statements was significantly associated with adherence, a finding important in informing future interventions aimed at improving adherence. Changes in the study populations, successes and failures of prescribers to provide accurate and free health care, and the challenging security context of the area are all possible explanations for the lack of an increase in adherence levels. Given all of these factors, sustaining the ACT adherence rates at the 2010 levels, through three years of insecurity is an achievement and suggests that if security can be improved barriers to improving health service quality and patient adherence to ACT could be removed.

## Abbreviations

ACT: Artemisinin-based combination therapy
AL: Artemether-lumefantrine
BCC: Behaviour change communication
IEC: Information education communication
IRS: Indoor residual spraying
KSH: Kenyan Shillings
LLIN: Long-lasting insecticidal nets
MoH: Ministry of Health
Q + T: Quinine and Tetracycline
RDT: Rapid diagnostic test
USD: United States Dollar
SP: Sulphadoxine-pyrimethamine
